# Genetic Diversity and Geographic Distribution of Cucurbit-Infecting Begomoviruses in the Philippines

**DOI:** 10.3390/plants12020272

**Published:** 2023-01-06

**Authors:** Zhuan Yi Neoh, Hsuan-Chun Lai, Chung-Cheng Lin, Patcharaporn Suwor, Wen-Shi Tsai

**Affiliations:** 1 Department of Plant Medicine, National Chiayi University, Chiayi City 600355, Taiwan; 2 Clover Seed Company Ltd., Hong Kong; 3 Agriculture Technology, King Mongkut’s Institute of Technology Ladkrabang, Bangkok 10520, Thailand

**Keywords:** leaf curl disease, *Squash leaf curl Philippines virus*, *Squash leaf curl China virus*, *Tomato leaf curl New Delhi virus*, Southeast Asia

## Abstract

Cucurbits are important economic crops worldwide. However, the cucurbit leaf curl disease (CuLCD), caused by whitefly-transmitted begomoviruses constrains their production. In Southeast Asia, three major begomoviruses, *Tomato leaf curl New Delhi virus* (ToLCNDV), *Squash leaf curl China virus* (SLCCNV) and *Squash leaf curl Philippines virus* (SLCuPV) are associated with CuLCD. SLCuPV and SLCCNV were identified in Luzon, the Philippines. Here, the genetic diversity and geographic distribution of CuLCD-associated begomoviruses in the Philippines were studied based on 103 begomovirus detected out of 249 cucurbit samples collected from 60 locations throughout the country in 2018 and 2019. The presence of SLCCNV and SLCuPV throughout the Philippines were confirmed by begomovirus PCR detection and viral DNA sequence analysis. SLCuPV was determined as a predominant CuLCD-associated begomovirus and grouped into two strains. Interestingly, SLCCNV was detected in pumpkin and bottle gourd without associated viral DNA-B and mixed-infected with SLCuPV. Furthermore, the pathogenicity of selected isolates of SLCCNV and SLCuPV was confirmed. The results provide virus genetic diversity associated with CuLCD for further disease management, especially in developing the disease-resistant cultivars in the Philippines as well as Southeast Asia.

## 1. Introduction

Cucurbit is an important economic crop cultivated worldwide [1]. In Southeast Asia, the harvested area of cucurbit crops has reached 240,603 ha with a production of more than 4,479,058 tonnes [2]. Meanwhile, cucurbit crops are also important in the Philippines with 31,454 ha harvested area and a production of 436,849 tonnes [2]. However, plant viral diseases affect the productivity and quality of cucurbit crops, causing significant economic losses [3,4]. More than 70 virus species have caused severe epidemic diseases in cucurbit production areas [5]. Cucurbit leaf curl disease (CuLCD) is one of the most important cucurbit viral diseases. The symptoms are a complex of leaf mosaic, yellowing, curling, enation, vein thickening and plant stunting [6,7,8]. This disease has constrained cucurbit crop production in many tropical and subtropical regions [9,10,11]. The disease incidence often reaches 100%, resulting in significant yield losses (up to 100%) [9,10,11]. CuLCD is caused by whitefly (*Bemisia tabaci*)-transmitted begomoviruses [7,8,9,10,11,12]. According to the ICTV taxonomic criteria for begomovirus, viruses with nucleotide identity < 91% of full-length DNA-A are considered as distinct species and those with 91–94% are considered as distinct strains of a virus species [13,14]. More than 17 begomoviruses were associated with CuLCD [14,15], and most of them are bipartite begomoviruses [14].

CuLCD has been reported in Southeast Asia, including Indonesia [16,17,18], Malaysia [15], Thailand [11,19], Philippines [20,21,22] and Vietnam [8,23]. Furthermore, cucurbit-infecting begomovirus sequences associated with CuLCD were also obtained from Cambodia and Laos [24]. Meanwhile, *Tomato leaf curl New Delhi virus* (ToLCNDV), *Squash leaf curl China virus* (SLCCNV) and *Squash leaf curl Philippines virus* (SLCuPV) are three major begomoviruses associated with CuLCD in Southeast Asia [15]. ToLCNDV was firstly identified in diseased tomato in northern India in 1995, and the host range (associated with more than 43 plant species) and geographical distribution are extending [25,26]. From 2012 to 2015, ToLCNDV was distributed to the Mediterranean and associated with cucurbit disease [26,27]. Between 2000 and 2017, various hosts were reported with ToLCNDV infection, such as carrot (*Daucus carota*), chilli pepper (*Capsicum annuum*), cotton (*Gossypium* spp.), okra (*Abelmoschus esculentus*), star gooseberry (*Sauropus androgynus*), etc. [24,26]. The wide host range and geographic distribution of ToLCNDV has constrained the cucurbit production in Southeast Asia [8,26,28]. So far, ToLCNDV has been reported to be associated with cucumber, luffa and melon in Indonesia [16,17,18,29], bitter gourd, bottle gourd, cucumber, oriental melon, squash, ridge gourd and wax gourd in Malaysia [15], and bottle gourd, cucumber, melon, luffa and star gooseberry in Thailand [11,19,30,31]. In addition, ToLCNDV sequences were also obtained from chilli pepper (JX416185) and wax gourd (MH328257) in Cambodia and from bitter gourd (MH328255) and luffa (MH328254) in Laos [24]. Furthermore, ToLCNDV is seed transmissible in zucchini and chayote [32,33]. SLCCNV was firstly identified on pumpkin in China in 1995 [34]. SLCCNV was associated with cucurbit disease in Vietnam in 2003 [23], and then in India, Pakistan, Thailand and the Philippines from 2001 to 2013 [22,35,36,37]. So far, in Southeast Asia, SLCCNV has been found on cucumber and squash in Indonesia [17,18], on bottle gourd and squash in Malaysia [15], on chayote in the Philippines [22], on pumpkin and wax gourd in Thailand [11,35] and on wax gourd and zucchini in Vietnam [23]. Furthermore, SLCCNV sequences were also obtained from wax gourd (MK064240) in Cambodia and pumpkin (KC857509) in Vietnam [24]. The squash disease associated virus, SLCuPV, was identified from diseased squash collected in 2001 and has become the predominant cucurbit-infecting begomovirus in Taiwan [15,38,39]. Afterwards, SLCuPV was reported in 2003 in the Philippines [21]. The distribution of SLCuPV is currently limited in the Philippines and Taiwan and associated with CuLCD in bottle gourd, chayote, pumpkin, wax gourd and zucchini [21,38,39].

Mixed infection of begomoviruses in host cells could accelerate genetic exchange through pseudorecombination and recombination of virus genomes [12,15]. Consequently, genetic exchange has resulted in the occurrence of novel begomoviruses, increased genetic variation, enhanced virulence, expanded host range, breakdown of host resistance and increased virus adaption to alternative host and environment [11,12,15,29,40]. Mixed infection of cucurbit-infecting begomoviruses has been reported in Southeast Asia and other countries in the world [10,11,15]. Mixed infection of *Squash leaf curl Yunnan virus* (SLCuYV) with SLCCNV or ToLCNDV was detected on pumpkin in Thailand [11]. Mixed infection of ToLCNDV with SLCCNV was observed on squash in Malaysia [15]. Based on both ToLCNDV and SLCCNV having bipartite genomic components (DNA-A and -B), the pseudorecombination of ToLCNDV DNA-A and SLCCNV DNA-B in nature field was also found in Malaysia [15]. The pseudorecombination of ToLCNDV DNA-A and SLCCNV DNA-B could alter host range and symptom severity [15]. Meanwhile, the recombination of ToLCNDV DNA-A and SLCCNV DNA-A was identified on melon in Indonesia [29] and on zucchini in India [40].

After CuLCD was observed on squash in the Philippines in 1997 [20], SLCuPV (formerly named SLCCNV-[Philippines]) was identified in Nueva Ecija, Luzon, in 2003 [21]. SLCCNV was also identified in Benguet, Luzon, in 2006 [22]. Both viruses were closely related by 90.3% sequence identity [21]. However, the study of CuLCD-associated begomovirus was limited in the Luzon area. Here, we investigated the genetic diversity and geographic distribution of cucurbit-infecting begomoviruses countrywide and conducted a pathogenicity study of the predominant virus to provide virus epidemiology for further CuLCD management in the Philippines as well as Southeast Asia.

## 2. Results

### 2.1. CuLCD Survey and Begomovirus Detection in the Philippines

Disease incidence was observed in bitter gourd (10–100%), bottle gourd (50–100%), chayote (20–100%), muskmelon (90–100%), pumpkin (50–100%), watermelon (50–100%) and wild melon (90–100%) (Table 1). Begomovirus DNA-A was detected in 103 out of 249 collected cucurbit samples using PCR with primer pair-PAL1v1978RYNN/PAR1c715H [15] (Table 1 and Table S1). Fifty-two out of 131 collected cucurbit samples were detected positive for begomovirus in the Luzon area. Thirteen out of 47 cucurbit samples collected from the Visayas area were detected as having begomovirus (Table 1). Moreover, begomovirus was also detected in 38 out of 71 cucurbit samples collected in Mindanao area. The begomovirus-detected samples included 62 pumpkins, 24 bottle gourds, 8 muskmelons, 6 chayotes and 3 wild melons (Table 1). All of the 83 bitter gourd samples and 35 watermelon samples were negative for begomovirus detection (Table 1). Viral DNA-B was also detected in all begomovirus-positive samples by PCR with primer pairs-SLCCNV-BV1/-BC1 (Table 1 and Table S1). However, virus-associated satellite DNA was not detected in all begomovirus-positive samples (data not shown).

### 2.2. Sequence Analysis of Cucurbit-Infecting Begomoviruses in the Philippines

Based on the results of begomovirus detection, the crops and locations of the samples collected, 55 begomoviral DNA-As and 50 DNA-Bs were partial sequenced (Table 1). Based on the sequence analysis, partial DNA-A sequences can be grouped into two Clusters (data not shown). Clusters 1 and 2 have the highest nucleotide identity with DNA-As of SLCuPV (>94.2%) and SLCCNV (>93.6%), respectively. The DNA-B partial sequences can also be grouped into two Clusters. Cluster 1 has the highest nucleotide identity (>90.2%) with SLCuPV DNA-Bs. Meanwhile, Cluster 2 has the highest nucleotide identity (>87.0%) with SLCCNV DNA-Bs.

Based on the results of partial sequence analysis, the locations of samples collected and the crops, 39 full-length DNA-A sequences and 38 full-length DNA-B sequences were obtained (Table 1). Conserved begomoviral nonanucleotide sequences (TAATATT/AC) in the stem loop structure of intergenic region (IR) were found in all sequences. Six open reading frames (ORFs AV1, AV2, AC1, AC2, AC3 and AC4) were determined in all 39 full-length DNA-A sequences. Meanwhile, two ORFs (BV1 and BC1) were also determined in all 38 full-length DNA-B sequences (Table S3).

**Figure 1:**
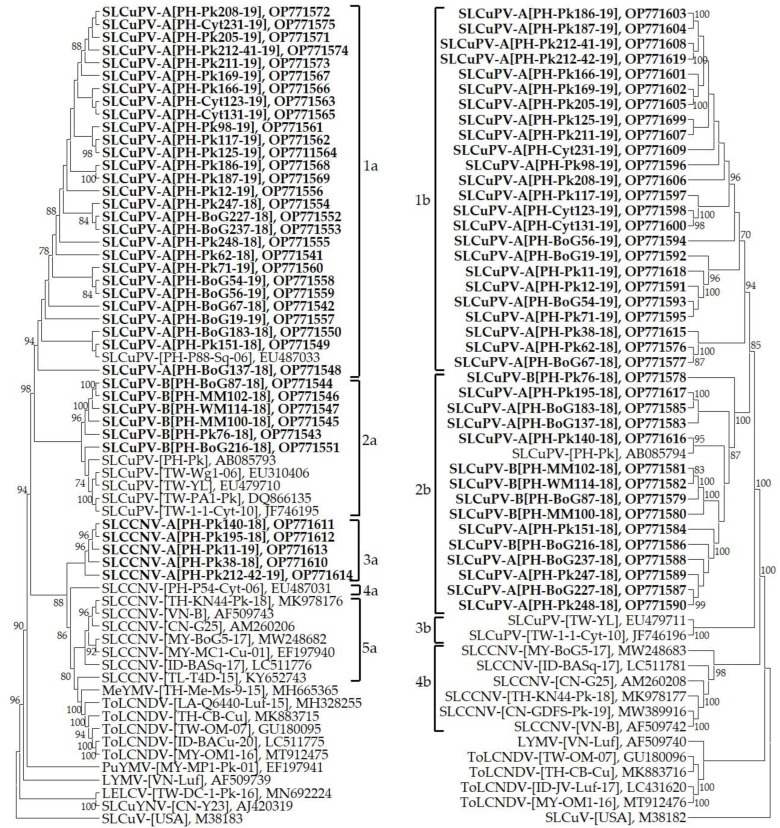
Phylogenic tree obtained from the alignment of complete DNA-A (**left**) and -B (**right**) nucleotide sequences of cucurbit-infecting begomoviruses. The sequences used for analysis including those newly identified in this study (in bold) and listed in Table S2. Both phylogenic trees were rooted on Squash leaf curl virus (SLCuV). The numbers of each branch indicated the percentage of 1000 bootstraps.

In the phylogenetic analysis, the full-length DNA-A sequences were grouped into five Clusters (Figure 1 and Table 2). According to the demarcation criteria of DNA-A nucleotide identity < 91.0% for begomovirus species, the newly identified virus isolates in Clusters 1a and 2a were considered as SLCuPV isolates (Figure 1 and Table 2). Their full-length DNA-A sequences revealed >91.2% identity with the reported SLCuPV isolates. Furthermore, isolates in Clusters 3a, 4a and 5a were SLCCNV and revealed 90.8–99.6% sequence identity (Figure 1 and Table 2). All newly identified SLCCNV isolates were in Cluster 3a.

Based on the demarcation criteria of DNA-A nucleotide identity < 94.0% for begomovirus strain in a species, SLCuPV isolates of Clusters 1a and 2a were composed of strains (A and B) (Figure 1 and Table 2). Virus isolates in Cluster 1a were SLCuPV strain A and shared sequence identity 94.4–99.9% to each other. The isolates of Cluster 2a composed of SLCuPV strain B showing 92.1–99.1% sequence identity to each other. However, isolates among SLCuPV strains A and B revealed sequence identity 91.2–96.1%. For newly identified SLCuPV isolates, isolates from eight bottle gourd, three of chayote and seventeen pumpkin samples were grouped in SLCuPV strain A, whereas SLCuPV strain B contained newly identified isolates from two bottle gourd, two muskmelon, one pumpkin and one wild melon samples. On the other hand, SLCCNV isolates were grouped into Clusters 3a, 4a and 5a and then also considered as distinct virus strains (Figure 1 and Table 2). The virus isolates in Cluster 3a were SLCCNV strain A which shared sequence identity > 95.9% with each other and 92.0–93.4% with other SLCCNV isolates. The virus isolate in Cluster 4a was considered as SLCCNV strain B showing sequence identity 90.8–93.4% with other SLCCNV isolates. Virus isolates in Cluster 5a were composed of SLCCNV strain C which showed sequence identity 93.2–99.6% to each other and 90.8–93.2% with other SLCCNV isolates. All newly identified SLCCNV isolates from pumpkin samples were grouped in SLCCNV strain A. The isolate SLCCNV-[PH-P54-Cyt-06] (EU487031) was clustered in SLCCNV strain B. The SLCCNV strain C composed of SLCCNV isolates that were reportedly from China, Indonesia, Malaysia, Thailand, Timor-Leste and Vietnam [11,15,17,18,23,24,35].

Based on the phylogenetic analysis, SLCuPV DNA-B sequences can be grouped into Clusters 1b, 2b and 3b (Figure 1 and Table 2). SLCuPV DNA-Bs in Cluster 1b shared 89.3–99.9% sequence identity and revealed <89.9% sequence identity with others. Virus DNA-B in Cluster 2b shared 85.5–98.6% sequence identity and showed 76.2–89.9% sequence identity with others. Both SLCuPV DNA-Bs in Cluster 3b revealed 96.7% sequence identity and showed <89.4% sequence identity with other DNA-Bs. The newly identified SLCuPV DNA-Bs from four bottle gourd, three chayote and sixteen pumpkin samples were grouped in Cluster 1b. Meanwhile, the newly identified SLCuPV DNA-Bs from six bottle gourd, two muskmelon, six pumpkin and one wild melon samples were in Cluster 2b. Interestingly, the DNA-Bs of SLCuPV-[TW-YL] (EU479711) and SLCuPV-[TW-1–1-Cyt-10] (JF746196) reported from Taiwan were in the distinct Cluster 3b. SLCCNV DNA-B sequences from China, Indonesia, Malaysia, Thailand and Vietnam were in Cluster 4b and revealed >72.8% sequence identity to each other.

### 2.3. Specific Detection of Cucurbit-Infecting Begomoviruses in the Philippines

Based on virus specific detection of virus infectious clones and selected field samples, the designed specific primer pairs could carry out the specific detection effectively: SLCuPV-1-SPAF/-1-SPAC for SLCuPV DNA-A, SLCCNV-1-SPAF/-1-SPAC for SLCCNV DNA-A, SLCCNV-BV1/SLCuPV-2-SPBC for SLCuPV DNA-B and SLCCNV-BV1/-1-SPBC and SLCCNV-3-SPBV/-3-SPBC for SLCCNV DNA-B (Figure S1 and Table S1). By the specific detection of all begomovirus-positive samples, the SLCuPV was detected as being the predominant cucurbit-infecting begomovirus in the Philippines (Table 1). The SLCuPV DNA-A and DNA-B were detected in all begomovirus-positive samples including bottle gourd, chayote, muskmelon, pumpkin and wild melon samples collected from Luzon, Visayas and Mindanao (Table 1). However, SLCCNV DNA-A was limited in twelve pumpkin samples collected in Luzon, six bottle gourd and seven pumpkin samples collected in Visayas and one pumpkin sample collected in Mindanao (Table 1). Interestingly, all of the SLCCNV DNA-A was detected as having mixed-infection with SLCuPV (Table 1). However, SLCCNV DNA-B was not present in all samples with SLCCNV DNA-A (Table 1).

### 2.4. Pathogenicity of Cucurbit-Infecting Begomoviruses in the Philippines

**Figure 2:**
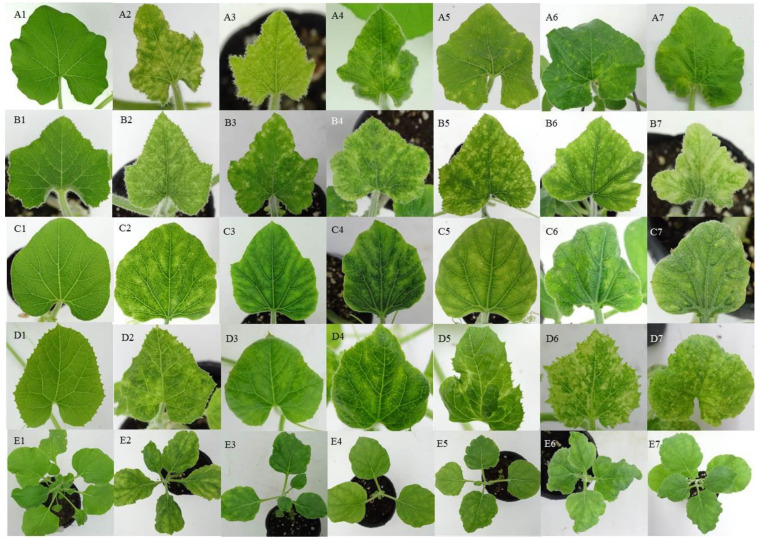
Symptoms on agroinoculated host plants with infectious DNAs cucurbit-infecting begomoviruses. Symptoms were induced by agroinoculation with *Agrobacterium tumefacies* LBA4404 that contained infectious DNA-A and DNA-B of SLCuPV-A[PH-BoG137-18] (**A2**–**E2**), -A[PH-Pk212-41-19] (**A3**–**E3**), -B[PH-Pk76-18] (**A4**–**E4**) and -B[PH-BoG216-18] (**A5**–**E5**). SLCCNV-A[PH-Pk195-18] DNA-A (**A6**–**E6**) and SLCCNV-A[PH-Pk212-42-19] DNA-A (**A7**–**E7**) were co-inoculated with a previous SCLCCNV-[MY-Sq3-5-16] DNA-B [15]. (**A1**–**E1**) were the symptomless plants, pumpkin (*Cucurbita maxima*), Chinese squash (*C. moschata*), bottle gourd (*Lagenaria siceraria*), melon (*Cucumis melo*) and tobacco (*Nicotiana benthamiana*) agroinoculated with pCAMBIA0380. Symptomatic plants, pumpkin (**A2**–**A7**), Chinese squash (**B2**–**B7**), bottle gourd (**C2**–**C7**), melon (**D2**–**D7**) and tobacco (**E2**–**E7**), were obtained after agroinoculation.

Based on the sequence analysis, four SLCuPV isolates (SLCuPV-A[PH-BoG137-18], -A[PH-Pk212-41-19], -B[PH-Pk76-18] and -B[PH-BoG216-18]), and two SLCCNV isolates (SLCCNV-A[PH-Pk195-18] and -A[PH-Pk212-42-19]) were selected for constructing the virus infectious clones. After the infectious DNA-A and DNA-B were co-inoculated, all virus infectious clones of SLCuPV-A[PH-BoG137-18], -A[PH-Pk212-41-19], -B[PH-Pk76-18] and -B[PH-BoG216-18] successfully infected bottle gourd, Chinese squash and pumpkin plants, and caused the disease symptoms of mosaic, yellow spot and leaf curling; furthermore, the presence of viral DNAs was also confirmed by PCR detection (Figure 2 and Table 3). The melon plants can be infected by SLCuPV-A[PH-BoG137-18], -B[PH-Pk76-18] and -B[PH-BoG216-18] with the disease symptoms of mosaic, yellow spot and leaf curling on inoculated plants. Virus infection was also confirmed by viral DNA detection. Furthermore, melon plants infected with SLCuPV-A[PH-Pk212-41-19] isolate were symptomless; however, the presence of virus DNAs was confirmed (Table 3). Tobacco plants infected with SLCuPV-A[PH-Pk212-41-19] and -B[PH-Pk76-18] were revealed to be symptomless, but viral DNAs were detected (Figure 2 and Table 3). Furthermore, tobacco plants infected with SLCuPV-B[PH-BoG216-18] revealed mild symptoms and viral DNAs were also detected (Figure 2 and Table 3). Meanwhile, symptoms of mosaic and leaf curling were observed on tobacco plants which were inoculated with SLCuPV-A[PH-BoG137-18] (Figure 2). Viral DNA-Bs were also detected in all inoculated plants which SLCuPV DNA-As detected. Since SLCCNV DNA-B was not present in the samples collected from the Philippines, the pathogenicity of SLCCNV isolates were tested by the co-agroinoculation of SLCCNV-[MY-Sq3-5-16] DNA-B with two infectious SLCCNV DNA-As. Both infectious SLCCNV isolates could successfully infect all cucurbit crops (Figure 2 and Table 3). Virus-infected plants revealed the disease symptoms of mosaic, yellow spot, leaf curling and yellowing. However, the mild symptoms of mosaic, leaf curling and yellowing were observed in the infected tobacco plants (Figure 2). The presence of viral DNAs was also confirmed by PCR detection (Table 3). When the SLCCNV DNA-A was co-inoculated with SLCuPV DNA-B from the same sample, both combinations of infectious clones could not infect cucurbit crops, while only tobacco plants could be infected (Table 3). The tobacco plants were revealed to be symptomless and only viral DNA-A was detected. In addition, the infectious viral DNA-As of SLCCNV and SLCuPV were also found to infect tobacco plants, but without developing symptoms. The presence of viral DNA-As were further confirmed by PCR detection (data not shown).

## 3. Discussion

Cucurbits are important crops and used in the human daily diet widely [1]. However, CuLCD caused by whitefly (*Bemisia tabaci*)-transmitted begomoviruses constrains cucurbit production and has resulted in significant yield losses up to 100% [9,10,11]. ToLCNDV, SLCCNV and SLCuPV are the predominant cucurbit-infecting begomoviruses in Southeast Asia [8,11,15,16,17,18,19,21,22,23,24,29,30,31,35,38,39]. Since CuLCD emerged in the Philippines as early as 1977 [20], SLCuPV and SLCCNV were identified in Luzon in 2003 and 2006, respectively [21,22]. SLCuPV was also identified in Taiwan in 2001 and then became an important cucurbit-infecting begomovirus [15,38,39]. Based on the samples collected during 2018–2019, SLCuPV and SLCCNV were confirmed as causes of CuLCD throughout the Philippines. SLCuPV was detected as a predominant virus. However, the virus detection results revealed that begomovirus was not detected in all cucurbits samples (103 out of 249). This may be due to the similar symptoms also induced by other cucurbit-infecting viruses such as cucumoviruses, tobamoviruses, poleroviruses and potyviruses [10,41,42,43,44]. In addition, a satellite was not detected in this study providing further evidence of neither SLCCNV nor SLCuPV associated with satellites in the Southeast Asia. So far, most satellites were associated with monopartite begomoviruses, rare with bipartite begomoviruses [45,46]. Meanwhile, a SLCCNV-associated *Croton yellow vein mosaic betasatellite* was detected on wax gourd from India [47], however, the relation of the satellite and SLCCNV should be further confirmed.

The high CuLCD incidence combined with high infection of begomovirus was detected in bottle gourd, chayote, muskmelon, pumpkin and wild melon samples, however begomovirus was not detected in watermelon and bitter gourd. This may be due to the limited samples surveyed in this study, or bitter gourd and watermelon might not be hosts of begomovirus in the Philippines. The predominant SLCuPV isolates in the Philippines were distinctly separated into two strains (A and B). Meanwhile, SLCuPV distribution was related to geographic area, with strain A distributed throughout the Philippines, whereas strain B was limited to the Luzon area. The SLCuPV-B was also distributed in Taiwan [38], the country north of the Philippines. A geographically related distribution has also been shown in a begomovirus study in Brazil [48]. Here, the geographically related distribution of SLCuPV strains suggests that the virus may originate from Luzon, where CuLCD was observed in 1977 [20]. The virus then evolved strains A and B which were distributed toward the south and north, respectively. The SLCCNV isolates from Southeast Asia and China were diverse with three distinct strains (A to C). Early in the century, SLCuPV was considered as a strain of SLCCNV based on both viruses revealing high sequence identity (90.3%) [21]. Our data also suggest SLCuPV could be evolved from SLCCNV (Figure 1). On the other hand, SLCCNV might have emerged in the Philippines earlier than SLCuPV. In addition, the distribution of SLCCNV was also related to the geographic area, the strains A and B were distributed in the Philippines, and the strain C was in China, Indonesia, Malaysia, Thailand, Timor-Leste and Vietnam. The geographically related distribution of SLCCNV was also confirmed in South Asia [40]. Further analysis of SLCCNV South Asian isolates indicated that they were also distinct to those from Southeast Asia and China (86.7–91.9% sequence identity). In the Philippines, the SLCCNV isolates obtained in this study were the strain A, whereas the previously reported isolate was the strain B. This implies that SLCCNV evolved in this period. The temporal evolution of begomoviruses was also studied in Brazil. The *Tomato severe rugose virus* (ToSRV) isolates obtained in 2004 and 2008 were in distinct groups [48]. However, further investigation should be conducted for monitoring of the temporal and spatial distribution of cucurbit-infecting begomoviruses in the Philippines as well as Southeast Asia.

Furthermore, the grouping of cucurbit-infecting begomoviral DNA-Bs was related to the viral DNA-As (Figure 1). However, cucurbit-infecting begomoviral DNA-Bs (71.5–99.9% sequence identity) were more diverse than DNA-As (85.2–99.9% sequence identity) in this study. This may result from the fact that both viral genetic components are facing different evolutionary patterns [48,49]. The accumulation level of DNA-B evolution can be higher than DNA-A and that has contributed to the high variation of the DNA-B population [48,50]. The reduction of the mutation and evolution rate in viral DNA-As may be the result of gene overlapping on the viral genome [49,51]. Thus, DNA-B can conduct greater variation; so far, no gene overlapping was observed in the viral component [48,49,51].

Based on the limited samples and virus specific detection, SLCuPV was determined as the predominant cucurbit-infecting begomovirus in the Philippines and has wider host range including bottle gourd, chayote, muskmelon, pumpkin and wild melon. The pathogenicity of predominant SLCuPV also revealed that all tested SLCuPV isolates can infect bottle gourd, Chinese squash, melon and pumpkin and revealed CuLCD symptoms as those reported previously [21]. In the Philippines, SLCCNV was only found in pumpkin and bottle gourd even when the virus was distributed countrywide. However, SLCCNV can infect more cucurbit crops than SLCuPV in Southeast Asia [8]. This may be due to the fact that SLCCNV was detected without viral DNA-B in the Philippines. The investigation was further confirmed by two SLCCNV infectious viral DNA-As, which could not infect bottle gourd, Chinese squash, melon and pumpkin, even when both viral DNA-As were co-inoculated with the SLCuPV DNA-B from same sample. However, when the infectivity of both SLCCNV DNA-As was completed by being co-inoculated with an infectious SLCCNV DNA-B [15], all infected cucurbits also revealed CuLCD symptoms. This implicated that SLCuPV is a more aggressive virus in the Philippines. However, the mixed infection of SLCuPV and SLCCNV provides the possibility for virus recombination, threatening cucurbit crops in the country.

In conclusion, based on the limited cucurbit samples collected in 2018–2019, two cucurbit-infecting begomoviruses, SLCCNV and SLCuPV were found throughout the Philippines. The varied SLCCNV and SLCuPV strains were of a temporal and spatial distribution, respectively. The SLCuPV was determined as predominant with a wide host range. Meanwhile, SLCCNV was detected without DNA-B and limited in bottle gourd and pumpkin. The pathogenicity of both SLCuPV strains was confirmed. The infectivity of SLCCNV DNA-A was also completed with a complementary viral DNA-B. Furthermore, the mixed infection of SLCuPV and SLCCNV implicates the possible recombination of both viruses. The results provided here are important for the development of virus-resistant cucurbit cultivars, and also strengthening the efficiency of CuLCD management in the Philippines as well as Southeast Asia. In addition, the developed virus specific primers can be applied for quarantine purposes. Furthermore, the infectious viral DNAs can also be applied in determining the virulence of viral DNAs, screening virus resistance, studying viral gene function and virus-vector-host interaction, etc. [52,53,54].

## 4. Materials and Methods

### 4.1. CuLCD Survey, Sample Collection and Viral DNA Extraction

Two hundred and forty-nine diseased samples from cucurbits including bitter gourd, bottle gourd, chayote, muskmelon, pumpkin, watermelon and wild melon (*Cucumis agristis*) with symptoms of mosaic, yellowing, leaf curling, blistering and stunting were collected from 60 locations throughout the Philippines during 2018 and 2019 (Table 1). The disease incidence in the field was investigated based on the estimated percentage of diseased plants observed in total. One hundred and thirty-one disease samples were collected on 7 cucurbit species from 29 locations in the north Philippines (Luzon). Forty-seven disease samples were collected on four cucurbit species from 11 locations in the central area of the Philippines (Visayas). Seventy-one diseased samples were also collected from four cucurbit species in 20 locations of Mindanao, the Philippines (Table 1). The samples were dried with silica gel and then preserved under −20 °C. For begomovirus detection, approximately 50 mg of dried leaf were used for viral DNA extraction with the modified Dellaporta method (modified from [55]).

### 4.2. Begomovirus Detection, Cloning and Sequence Analysis of Begomoviral DNAs

Begomoviral DNA-A was detected by PCR with degenerate primer pair-PAL1v1978RYNN/PAR1c715H with annealing temperature 58 °C [15] (Table S1). Furthermore, the viral DNA-Bs were detected with degenerate primer pair-SLCCNV-BV1/BC1 with annealing temperature 48 °C (Table S1). The virus-associated satellite DNA was also detected by primer pair-Beta01/Beta02 [56]. The PCR reaction mixture contained 5 μL of extracted DNA, 0.5 μL each of 10 mM forward and reverse primers, 12.5 μL of 2X Hieff^TM^ PCR Master mix (Yeasen, China) and adjudged the volume to 25 μL by deionized water. The PCR was conducted using PCR thermal cycle (Applied Biosystems, Thermo Fisher Scientific, USA). The PCR amplification for viral DNA-A detection was conducted for 30 cycles of denaturation at 94 °C for 1 min, annealing at annealing temperature of primers for 1 min and extension at 72 °C for 1.5 min, and followed by a final extension at 72 °C for 10 min. The expected amplicon for DNA-A detection was approximately 1.5 kb in size. The PCR amplification for viral DNA-B detection was the same as for viral DNA-A, with an extension time of 0.5 min, and amplicon was approximately 0.5 kb in size.

Full-length viral DNA was amplified by PCR with abutting primer pair (Table S1). Q5 High-Fidelity DNA Polymerase (New England BioLabs, Ipswich, MA, USA) was used in PCR amplification. The amplified full-length viral DNAs were purified by the Promega Wizard^®^ SV Gel and PCR Clean-up System (Promega, Madison, WI, USA). The purified viral DNAs were cloned by pGEM^®^-T Easy Vector System I (Promega, USA) and sequenced automatically (Genomics, New Taipei City, Taiwan). The related sequences of begomoviral DNAs were also retrieved from GenBank, NCBI for phylogenic analysis (Table S2). Multiple alignment and nucleotide sequence identity of viral full-length sequences were generated using DNASTAR software (DNASTAR, USA). Phylogenic tree was conducted using Molecular Evolutionary Genetics Analysis X (MEGA X) software using the Muscle alignment and the Maximum Likelihood algorithm with 1000 bootstrap replications [57]. Following verification of the begomovirus ORFs, viral DNA sequences were submitted to GenBank, NCBI (National Center for Biotechnology Information).

### 4.3. Specific Detection of Begomoviral DNAs

Based on the sequence analysis results from the Philippines samples and sequences retrieved from GenBank, NCBI (Table S2), primer pairs were designed for the specific detection of SLCuPV and SLCCNV by PCR (Figure S1 and Table S1). The primer pair-SLCuPV-1-SPAF/-1-SPAC (annealing temperature 47.5 °C) was specific for SLCuPV DNA-A with amplicon in 0.85 kb. The primer pair-SLCCNV-1-SPAF/-1-SPAC (annealing temperature 48.5 °C) was designed for the specific detection of SLCCNV DNA-A with an amplicon approximately in 0.75 kb. The primer pair-SLCCNV-BV1/SLCuPV-2-SPBC (annealing temperature 47 °C) was also used for the specific detection of SLCuPV DNA-B, with the expected amplicon of 0.35 kb in size. For the specific detection of SLCCNV DNA-B, two primer pairs, SLCCNV-BV1/-1-SPBC (annealing temperature 37.5 °C) and SLCCNV-3-SPBV/-3-SPBC (annealing temperature 46 °C) were designed with an amplicon approximately of 1.5 kb and 0.5 kb, respectively.

### 4.4. Construction and Agroinoculation of Begomoviral Infectious DNAs

Based on the sequence analysis results, four SLCuPV isolates and two SLCCNV isolates were selected for development of infectious clones. For the infectious DNA-As of SLCuPV-A[PH-BoG137-18], -A[PH-Pk212-41-19], -B[PH-Pk76-18] and -B[PH-BoG216-18], partial viral DNA-A was released from recombinant plasmids with *Sal* I and *Bam* HI digestion (0.23 mer) and inserted into the binary vector pCAMBIA0380 (AF234290) [58]. Consequently, the infectious SLCuPV DNA-A was generated using head-tail ligation of full-length DNA-A, which was released by *Bam* HI digestion. For the infectious DNA-Bs of SLCuPV-A[PH-BoG137-18] and -B[PH-BoG216-18], partial DNA-B was also released by *Sal* I and *Nco* I digestion (0.61 and 0.62 mer, respectively) and then inserted into the pCAMBIA0380. For infectious DNA-Bs of SLCuPV-A[PH-Pk212-41-19] and -B[PH-Pk76-18], partial DNA-B was also released by *Eco* RI and *Nco* I digestion (0.58 mer) and inserted into the pCAMBIA0380. The infectious SLCuPV DNA-Bs were constructed by head-tail ligation of full-length DNA-B which was released by *Nco* I digestion. For infectious DNA-Bs of SLCuPV-A[PH-Pk195-18] and -A[PH-Pk212-42-19], partial DNA-B was released by *Bgl* II and *Hpa* I digestion (0.59 mer) and inserted into the pCAMBIA0380. Both infectious DNA-Bs were generated by a head-tail ligation of full-length DNA-B which was released by *Hpa* I digestion. For infectious DNA-A of SLCCNV-A[PH-Pk195-18], partial viral DNA-A was obtained using *Eco* RI and *Bam* HI digestion (0.29 mer) and then cloned into the pCAMBIA0380. For the infectious DNA-A of SLCCNV-A[PH-Pk212-42-19], partial viral DNA-A was released from recombinant plasmid by *Apa* I and *Bam* HI digestion (0.4 mer) and cloned into pCAMBIA0380. The infectious SLCCNV DNA-As were generated by a head-tail ligation of full-length DNA-A which was released by *Bam* HI digestion. All infectious viral DNAs were transformed into *Agrobacterium tumefaciens* LBA4404. The transformed *A. tumefaciens* LBA4404 with viral infectious DNA was cultured in YEP broth (10 g peptone, 5 g yeast extract and 5 g sodium chloride per liter) containing 50 μg/mL Streptomycin and 50 μg/mL Kanamycin at 28 °C with 200 rpm of shaking for 48 h. Following the concentration of bacterial cultures adjusted to OD600_nm_ = 1, the agrobacterial cultures of infectious viral DNA-A and DNA-B were mixed well with a ratio of 1:1. Two hundred microliters of the culture was inoculated to the stems and petioles of the cucurbit plant in three to five true leaf stage. The bottle gourd HV-026 (*Lagenaria siceraria cv.* Ever Happiness), pumpkin HV-369 (*Cucurbita maxima*) and oriental melon (*Cucumis melo cv.* Honey world) were obtained from Known-You Seed Co., LTD., Taiwan. Chinese squash farmers (*Cucurbita moschata*) were also used. Tobacco plants in the four to six leaf stage (*Nicotiana benthamiana*) were also agroinoculated. The concentration of agrobacteria containing viral DNA-A alone was also adjusted to OD_600nm_ = 1 and inoculated to testing plants. Those inoculated plants were cultured in an insect-free greenhouse at 25 °C. The symptom development was observed and recorded for 28 days, and then the presence of viral DNAs was also detected by PCR with primer pair-PAL1v1978RYNN/PAR1c715H for DNA-A and primer pair-SLCCNV-BV1/BC1 for DNA-B. Since SLCCNV DNA-B was not present in the samples collected from the Philippines, the pathogenicity of SLCCNV isolates was tested by co-inoculating their DNA-As with SLCCNV-[MY-Sq3-5-16] DNA-B (MW248681) previously developed [15].

## Figures and Tables

**Table 1:** Begomovirus detection of cucurbit samples collected in the Philippines.

Locations	Year of Collection	Crops	Fields	Disease Incidence, (%)	Collected Samples	BGV Detected		Partial Sequences		Full Length Sequences			Specific Detection of Viral DNAs			
						DNA-A	DNA-B	DNA-A	DNA-B	SLCuPV		SLCCNV	SLCuPV		SLCCNV	
										DNA-A	DNA-B	DNA-A	DNA-A	DNA-B	DNA-A	DNA-B
Luzon	2018	Bitter gourd	12	30–100	39	0	0	0	0	0	0	0	0	0	0	0
		Bottle gourd	11	50–100	36	18	18	10	9	7	7	0	18	18	0	0
		Chayote	1	90–100	2	0	0	0	0	0	0	0	0	0	0	0
		Muskmelon	1	90–100	8	8	8	2	2	2	2	0	8	8	0	0
		Pumpkin	11	80–100	29	23	23	14	12	5	8	3	23	23	12	0
		Watermelon	4	90–100	14	0	0	0	0	0	0	0	0	0	0	0
		Wild melon	1	90–100	3	3	3	1	1	1	1	0	3	3	0	0
Visayas	2019	Bitter gourd	5	30–100	22	0	0	0	0	0	0	0	0	0	0	0
		Bottle gourd	2	90–100	7	6	6	5	4	3	3	0	6	6	6	0
		Pumpkin	2	90–100	7	7	7	5	5	2	3	1	7	7	7	0
		Watermelon	3	90–100	11	0	0	0	0	0	0	0	0	0	0	0
Mindanao	2019	Bitter gourd	6	10–80	22	0	0	0	0	0	0	0	0	0	0	0
		Chayote	3	20–100	7	6	6	3	3	3	3	0	6	6	0	0
		Pumpkin	9	50–100	32	32	32	15	14	11	11	1	32	32	1	0
		Watermelon	2	50–100	10	0	0	0	0	0	0	0	0	0	0	0
		**Total**	**60**		**249**	**103**	**103**	**55**	**50**	**34**	**38**	**5**	**103**	**103**	**26**	**0**

**Table 2:** Nucleotide sequence identity (%) among DNA sequences of cucurbit-infecting begomoviruses in the Philippines.

Virus Cluster ^a^	Virus species ^b^	Cluster 1a	Cluster 2a	Cluster 3a	Cluster 4a	Cluster 5a
Cluster 1a	SLCuPV	94.4–99.9				
Cluster 2a	SLCuPV	91.2–96.1	92.1–99.1			
Cluster 3a	SLCCNV	88.9–91.4	87.8–91.4	95.9–98.8		
Cluster 4a	SLCCNV	86.6–87.8	86.2–88.0	93.0–93.4	100	
Cluster 5a	SLCCNV	85.9–88.3	85.2–87.9	92.0–93.2	90.8–92.6	93.2–99.6
		Cluster 1b	Cluster 2b	Cluster 3b	Cluster 4b	
Cluster 1b	SLCuPV	89.3–99.9				
Cluster 2b	SLCuPV	81.4–89.9	85.5–98.6			
Cluster 3b	SLCuPV	85.0–88.6	76.2–89.4	96.7		
Cluster 4b	SLCCNV	74.3–81.1	71.5–81.1	74.1–78.6	72.8–98.0	

^a^ Based on the phylogenetic trees of DNA-A and DNA-B in Figure 1. ^b^ SLCuPV: *Squash leaf curl Philippines virus*; SLCCNV: *Squash leaf curl China virus*.

**Table 3:** Pathogenicity of SLCuPV infectious clones from the Philippines.

Infectious Clones	Bottle Gourd		Melon		Pumpkin		Chinese Squash		Tobacco	
	Symptom ^a^	PCR ^b^	Symptom	PCR	Symptom	PCR	Symptom	PCR	Symptom	PCR
SLCuPV-A[PH-BoG137-18] DNA-A + -B	5/6	5/6	1/2	1/2	5/5	5/5	5/8	5/8	7/9	9/9
SLCuPV-A[PH-Pk212-41-19] DNA-A + -B	4/4	4/4	0/4	1/4	2/4	3/4	2/4	3/4	0/6	6/6
SLCuPV-B[PH-Pk76-18] DNA-A + -B	7/8	7/8	2/5	3/5	4/9	4/9	5/6	5/6	0/11	9/11
SLCuPV-B[PH-BoG216-18] DNA-A + -B	6/6	6/6	1/4	1/4	6/6	6/6	6/8	6/8	1/4	4/4
SLCCNV-A[PH-Pk195-18] DNA-A + SLCCNV-[MY-Sq3-5-16] DNA-B ^c^	3/3	3/3	1/1	1/1	3/3	3/3	3/3	3/3	2/2	2/2
SLCCNV-A[PH-Pk195-18] DNA-A + SLCuPV-A[PH-Pk195-18] DNA-B	0/2	0/2	0/5	0/5	0/7	0/7	0/3	0/3	0/6	6/6 ^d^
SLCCNV-A[PH-Pk212-42-18] DNA-A + SLCCNV-[MY-Sq3-5-16] DNA-B ^c^	3/3	3/3	1/2	1/2	4/4	4/4	3/3	3/3	1/1	1/1
SLCCNV-A[PH-Pk212-42-19] DNA-A + SLCuPV-A[PH-Pk212-42-19] DNA-B	0/6	0/6	0/4	0/4	0/5	0/5	0/6	0/6	0/10	10/10 ^d^

^a^ Symptomatic plants/inoculated plants were observed at 28 days after agroinoculation. ^b^ Viral DNA-A- and DNA-B-detected plants/inoculated plants were determined at 28 days after agroinoculation. ^c^ The infectious SLCCNV-[MY-Sq3-5-16] DNA-B was developed previously [15]. ^d^ Viral DNA-A-detected plants/inoculated plants were determined at 28 days after agroinoculation. All plants were detected as having viral DNA-A present, but no DNA-B was detected.

## Data Availability

Not applicable.

## Supplementary Materials

The following are available online at https://www.mdpi.com/article/10.3390/plants12020272/s1, Table S1: Sequences of primers used in this study, Table S2: The sequences of begomoviral DNAs used in this study, Table S3: Sequence characteristics of cucurbit-infecting begomovirus DNAs identified in this study, Figure S1: 1.2% agarose gel electrophoresis of specific detection of cucurbit-infecting begomoviruses in the Philippines.
