# Supplementary material for: Genetic Diversity and Geographic Distribution of Cucurbit-Infecting Begomoviruses in the Philippines
Source: Plants (Basel). 2023 Jan 6;12(2):272. doi: 10.3390/plants12020272 (PMC9862860; doi:10.3390/plants12020272)
Supplement: Supplementary file 1 [file plants-12-00272-s001.zip › plants-2065094-supplementary.pdf]

plants

MDPI

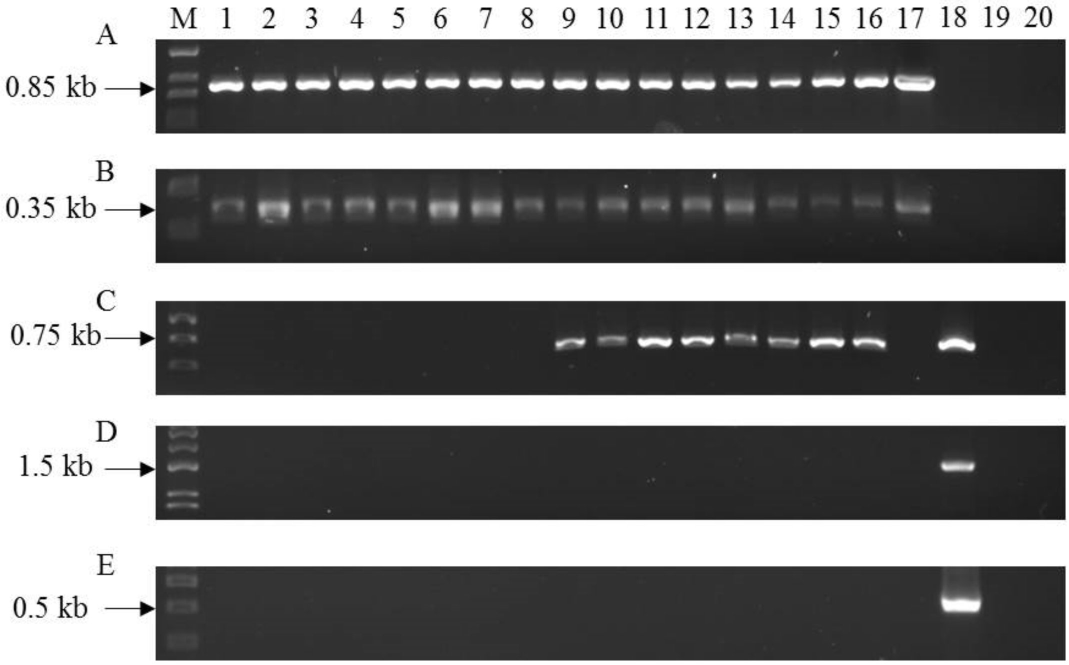

Figure S1. 1.2% agarose gel electrophoresis of specific detection of cucurbit-infecting begomoviruses in the Philippines. The virus specific detection was conducted by PCR using the *Squash leaf curl Philippines virus* (SLCuPV) DNA-A specific primer pair-SLCuPV-1-SPAF/-1-SPAC (A), SLCuPV DNA-B specific primer pair-SLCCNV-BV1/SLCuPV-2-SPBC (B), *Squash leaf curl China virus* (SLCCNV) DNA-A specific primer pair-SLCCNV-1-SPAF/-1-SPAC (C), the SLCCNV DNA-B specific primer pair-SLCCNV-BV1/-1-SPBC (D) and SLCCNV-3-SPBV/-3-SPBC (E). Lane M is the DNA molecular 1kb size marker. Lanes 1 to 16 are viral DNAs extracted from symptomatic cucurbit leaf samples Pk76, MM100, WM114, BoG216, Pk98, Cyt131, Pk187, Cyt231, Pk38, Pk129, Pk195, Pk247, BoG18, BoG56, Pk71, Pk212. Lane 17 is viral DNA extracted from a SLCuPV infected pumpkin plant. Lane 18 is viral DNA extracted from a SLCCNV infected pumpkin plant. Lane 19 is a healthy squash. Lane 20 is a buffer control.

Table S1. Sequences of primers used in this study.

**Primers**	**Sequence (5′ to 3′)[a]**	**Purpose**
PAL1v1978RYNN[b]	GCATCTGCAGGCCCACRYNGTYTTNCCNGT	General detection of begomoviruses DNA-A
PAR1c715H[b]	GATTTCTGCAGTTDATRTTHTCRTCCATCCA	General detection of begomoviruses DNA-A
SLCCNV-BV1[b]	GTAATGYTAAATTACATTGG	General detection of DNA-Bs of SLCuPV and SLCCNV
SLCCNV-BC1[b]	TYVACKGACGTCGAATTCCGA	General detection of DNA-Bs of SLCuPV and SLCCNV
19PH71-FAV	CGCGGATCCACTGTTGCACGAAT	Full-length DNA-A amplification of 18PH67, 18PH76, 18PH151, 18PH183, 18PH195, 18PH216, 18PH227, 18PH247, 19PH12, 19PH54, 19PH55, 19PH56, 19PH70, 19PH71, 19PH98, 19PH117, 19PH118, 19PH123, 19PH125, 19PH166, 19PH169, 19PH171, 19PH172, 19PH186, 19PH205,19PH208, 19PH211
18PH114-FAV	CACGGATCCACTACTGCACGA	Full-length DNA-A amplification of 18PH114
19PH131-FAV	CGAGGATCCACTGTTACACGAAT	Full-length DNA-A amplification of 19PH131
19PH11-FAV	CATGGATCCACTGCTGCACGA	Full-length DNA-A amplification of 18PH38, 18PH62, 18PH140, 18PH158, 19PH10, 19PH11, 19PH19, 19PH54
19PH117-FAC	GTAGGATCCCACATGTTGTGGATC	Full-length DNA-A amplification of 18PH67, 18PH76, 18PH151, 18PH183, 18PH195, 18PH216, 18PH227, 18PH247, 19PH12, 19PH54, 19PH55, 19PH70, 19PH71, 19PH98, 19PH117, 19PH118, 19PH125, 19PH131, 19PH166, 19PH169, 19PH171, 19PH172, 19PH186, 19PH205,19PH208, 19PH211, 19PH231
18PH114-FAC	GTAGGATCCCACATGGTGTGAATC	Full-length DNA-A amplification of 18PH114
19PH56-FAC	GAAGGATCCCACATGTTGTGGATAC	Full-length DNA-A amplification of 19PH56
19PH123-FAC	CTAGGATCCCACATGTTGTGGAGC	Full-length DNA-A amplification of 19PH123
18PH38-FAC	GTAGGATCCCACATGTTCTGGAC	Full-length DNA-A amplification of 18PH38, 18PH62, 18PH140, 18PH158, 19PH10, 19PH11, 19PH19, 19PH54
18PH247-5 FAV1	GACAATGAACCGAGCACTGCA	For completing DNA-A sequencing of 18PH38, 18PH62, 18PH67, 18PH76, 18PH87, 18PH114, 18PH137, 18PH140, 18PH151, 18PH183, 18PH195, 18PH216, 18PH227, 18PH237, 18PH247, 19PH11, 19PH19, 19PH54, 19PH56, 19PH71, 19PH98, 19PH117, 19PH123, 19PH125, 19PH131, 19PH166, 19PH169, 19PH186, 19PH205, 19PH208, 19PH211, 19PH231
18PH247-5 FAC1	CACATTTCCATCCGAACGTTCA	For completing DNA-A sequencing of 18PH227, 18PH237, 18PH247, 19PH54, 19PH98, 19PH117, 19PH123, 19PH125, 19PH131, 19PH166, 19PH169, 19PH186, 19PH205, 19PH208, 19PH211, 19PH231
18PH216-8 FAC1	CACATTTCCATCCGAACATTCA	For completing DNA-A sequencing of 18PH38, 18PH62, 18PH67, 18PH76, 18PH87, 18PH114, 18PH137, 18PH140, 18PH151, 18PH183, 18PH195, 18PH216, 19PH11, 19PH19, 19PH56, 19PH71
PKB-V	GCTCCATGGATTGATGCGTTATCG	Full-length DNA-B amplification of 18PH62, 18PH67, 18PH76, 18PH87, 18PH100, 18PH102, 18PH114, 18PH137, 18PH151, 18PH183, 18PH216, 18PH227, 18PH237, 18PH247 ,18PH248, 19PH12, 19PH19, 19PH54, 19PH56, 19PH71, 19PH98, 19PH117, 19PH123,19PH125, 19PH131, 19PH166, 19PH169, 19PH186, 19PH187, 19PH205, 19PH208, 19PH211, 19PH212, 19PH231
PKPHB-C	GTACCATGGTATTTCCGAGATCTG	Full-length DNA-B amplification of 18PH62, 18PH67, 18PH76, 18PH87, 18PH137, 18PH151, 18PH237, 19PH12, 19PH19, 19PH54, 19PH56, 19PH71, 19PH117, 19PH125, 19PH131, 19PH166, 19PH169, 19PH205, 19PH208
PKTWB-C	CGTCCATGGCATTTCCGAGATC	Full-length DNA-B amplification of 18PH100, 18PH102, 18PH114, 18PH183, 18PH216, 18PH227, 28PH247, 18PH248, 19PH98, 19PH123, 19PH186, 19PH187, 19PH211, 19PH212, 19PH231
19PH11-FBV	CGCGTTAACAGTGAATTCTTTGCTC	Full-length DNA-B amplification of 18PH38, 18PH195, 19PH11
18PH129-FBC	CGCGTTAACGACATAGATTTCTGGTC	Full-length DNA-B amplification of 18PH38, 18PH195

19PH11-FBC	CGCGTTAACGACATCGATTTCTGGTC	Full-length DNA-B amplification of 19PH11
18PH76-2 FBV1	GTATGGTTAAGCGATGTACCT	For completing DNA-B sequencing of 18PH62, 18PH67, 18PH76, 18PH137, 18PH151, 18PH216, 18PH227, 18PH237, 19PH56, 19PH98, 19PH117, 19PH123, 19PH125, 19PH131, 19PH166, 19PH169, 19PH186, 19PH205, 19PH208, 19PH211
18PH114-7 FBV1	GTATGGTTGAGCGATGTACCT	For completing DNA-B sequencing of 18PH87, 18PH114
19PH71-7 FBV1	GTATGGTTAAGTGATGTACCT	For completing DNA-B sequencing of 19PH19, 19PH54, 19PH71
19PH231-4 FBV1	GTATGGTTAAGCGATATACCT	For completing DNA-B sequencing of 19PH231
18PH100-13 FBV1	AATTACATTGGTTAGTGTAC	For completing DNA-B sequencing of 18PH100, 18PH102
19PH205-9 FBV2	GTAATGCAAATTACAGTTGCATT	For completing DNA-B sequencing of 19PH169, 19PH205
19PH56-6 FBV2	GTAATGCAAATTGCAGTTGCATC	For completing DNA-B sequencing of 18PH76, 18PH87, 18PH114, 18PH137, 18PH151, 18PH216, 18PH227, 28PH237, 19PH56
19PH19-7 FBV2	GACTGCATTTATTGGTACTT	For completing DNA-B sequencing of 19PH19, 19PH54, 19PH71, 19PH125
18PH62-9 FBV2	GACTGCATTGATTGGTACTT	For completing DNA-B sequencing of 18PH62, 18PH67, 19PH56, 19PH98, 19PH117, 19PH123, 19PH131, 19PH166, 19PH186, 19PH208, 19PH231
18PH38-7 FBV1	ACGTGCCATGTACTCAGA	For completing DNA-B sequencing of 18PH38
18PH129-4 FBV1	ACATGCCATGTACTCAGA	For completing DNA-B sequencing of 18PH195
19PH11-2 FBV1	ACCTATCATGTATCCAGA	For completing DNA-B sequencing of 19PH11
19PH212-17 FBV1	ACCTATCATGTATTCAGA	For completing DNA-B sequencing of 19PH212
18PH100-FBC1	TGCCATTTAGAGACATACTA	For completing DNA-B sequencing of 18PH100, 18PH102
18PH38-7 FBC1	CACGACAGGCACTAACAG	For completing DNA-B sequencing of 18PH38, 18PH195, 19PH11
19PH212-17 FBC1	CACGACAGGAACTAACAG	For completing DNA-B sequencing of 19PH212
CAMBIA-V	TAATAACACATTGCGGACGT	For confirming the infectious clones
CAMBIA-C	AATGTTTGAACGATCGGGGA	For confirming the infectious clones
SLCuPV-1-SPAC	GCTTTAKMWATAATWARRGARGAACT	Specific detection of SLCuPV DNA-A
SLCuPV-1-SPAF	CAGGTTGTRGTTGAACATGATG	Specific detection of SLCuPV DNA-A
SLCCNV-1-SPAC	GCNTTRAARGTRYTRARRGAATTAGC	Specific detection of SLCCNV DNA-A
SLCCNV-1-SPAF	ARATCAATDCGTCKDCGYCTG	Specific detection of SLCCNV DNA-A
SLCuPV-2-SPBC	CAYAGTTCAAGATCVTTGC	Specific detection of SLCuPV DNA-B
SLCCNV-1-SPBC	CTMTAWRGTATWTATAAGAC	Specific detection of SLCCNV DNA-B
SLCCNV-3-SPBV	GDGTCTTATAWATACCTT	Specific detection of SLCCNV DNA-B
SLCCNV-3-SPBC	RTCGTCTYSCHGGAGTAT	Specific detection of SLCCNV DNA-B

[a]B=C, G, T; D=A, G, T; H=A, C, T; K=G, T; M=A, C; N=A, T, G, C; R=A, G; S=C, G; V=A, C, G; W=A, T; Y=C, T.
[b]Primers were published in [15].

Table S2. The sequences of begomoviral DNAs used in this study.

Viruses	Abbreviation	GenBank accession	
		DNA-A	DNA-B
Squash leaf curl China virus pumpkin isolate from Thailand	SLCCNV-[TH]	AB330078	
Squash leaf curl China virus B isolate of cucurbits from Vietnam	SLCCNV-[VN-B]	AF509743	AF509742
Squash leaf curl China virus Hn61 isolate of squash from China	SLCCNV-[CN-Hn61-Sq]	AM260205	AM260207
Squash leaf curl China virus G25 isolate of squash from China	SLCCNV-[CN-G25]	AM260206	AM260208
Squash leaf curl China virus MC1 isolate of cucumber from Malaysia	SLCCNV-[MY-MC1-Cu-01]	EF197940	
Squash leaf curl China virus P54 isolate of chayote from Philippines	SLCCNV-[PH-P54-Cyt-06]	EU487031	
Squash leaf curl China virus melon isolate from China	SLCCNV-[CN-Me-10]	HM566112	HM566113
Squash leaf curl China virus GZ01 isolate of squash from China	SLCCNV-[CN-GZ01-Sq-11]	KC171648	KC171649
Squash leaf curl China virus Hanoi isolate of squash from Vietnam	SLCCNV-[VN-Hanoi-Sq-12]	KC857509	KC857510
Squash leaf curl China virus SY isolate of squash from China	SLCCNV-[CN-SY-Sq-13]	KF999983	KF999984
Squash leaf curl China virus T4D isolate of pumpkin from Timor-Leste	SLCCNV-[TL-T4D-15]	KY652743	
Squash leaf curl China virus squash isolate from Indonesia	SLCCNV-[ID-BASq-17]	LC511776	LC511781
Squash leaf curl China virus Guangxi2017 isolate of Squash from China	SLCCNV-[CN-Guangxi2017-Sq-17]	MG525551	MG525552
Squash leaf curl China virus KN44 isolate of pumpkin from Thailand	SLCCNV-[TH-KN44-Pk-18]	MK978176	MK978177
Squash leaf curl China virus YN4560 isolate of pumpkin from China	SLCCNV-[CN-YN4560-Pk-14]	MN218672	
Squash leaf curl China virus KN42A isolate of pumpkin from Thailand	SLCCNV-[TH-KN42A-Pk-16]	MN365018	
Squash leaf curl China virus KN52A isolate of pumpkin from Thailand	SLCCNV-[TH-KN52A-Pk-16]	MN437659	
Squash leaf curl China virus 16MY3 isolate of squash from Malaysia	SLCCNV-[MY-Sq3-5-16]	MW248679	MW248668
Squash leaf curl China virus 16MY5 isolate of bottle gourd from Malaysia	SLCCNV-[MY-BoG5-17]	MW248682	MW248683
Squash leaf curl China virus 17MY107 isolate of squash from Malaysia	SLCCNV-[MY-Sq107-17]	MW248685	MW248686
Squash leaf curl China virus 17MY115 isolate of squash from Malaysia	SLCCNV-[MY-Sq115-17]	MW248687	MW248688
Squash leaf curl China virus 17MY157 isolate of squash from Malaysia	SLCCNV-[MY-Sq157-17]	MW248689	MW248690
Squash leaf curl China virus GDFS isolate of pumpkin from China	SLCCNV-[CN-GDFS-Pk-19]	MW389915	MW389916
Squash leaf curl Philippines virus squash isolate from Philippines	SLCuPV-[PH-Pk]	AB085793	AB085794
Squash leaf curl Philippines virus PA1 isolate of pumpkin from Taiwan	SLCuPV-[TW-PA1-Pk]	DQ866135	
Squash leaf curl Philippines virus Wg1 isolate of wax gourd from Taiwan	SLCuPV-[TW-Wg1-06]	EU310406	
Squash leaf curl Philippines virus YL isolate from Taiwan	SLCuPV-[TW-YL]	EU479710	EU479711
Squash leaf curl Philippines virus P88 isolate of squash from Philippines	SLCuPV-[PH-P88-Sq-06]	EU487033	
Squash leaf curl Philippines virus 1-1 isolate of chayote from Taiwan	SLCuPV-[TW-1-1-Cyt-10]	JF746195	JF746196

Squash leaf curl Yunnan virus Y23 isolate of cucurbit from China	SLCuYNV-[CN-Y23]	AJ420319	
Tomato leaf curl New Delhi virus melon isolate from Taiwan	ToLCNDV-[TW-OM-07]	GU180095	GU180096
Tomato leaf curl New Delhi virus cucumber isolate from Indonesia	ToLCNDV-[ID-BACu-20]	LC511775	LC511780
Tomato leaf curl New Delhi virus luffa isolate from Indonesia	ToLCNDV-[ID-JV-Luf-17]	LC431619	LC431620
Tomato leaf curl New Delhi virus luffa isolate from Laos	ToLCNDV-[LA-Q6440-Luf-15]	MH328254	
Tomato leaf curl New Delhi virus cucumber isolate from Thailand	ToLCNDV-[TH-CB-Cu]	MK883715	MK883716
Tomato leaf curl New Delhi virus 16MY1 isolate of oriental melon from Malaysia	ToLCNDV-[MY-OM1-16]	MT912475	MT912476
Melon yellow mosaic virus Me-MS-9 isolate of melon from Thailand	MeYMV-[TH-Me-Ms-9-15]	MH665365	
Lisianthus enation leaf curl virus DC-1 isolate of pumpkin from Taiwan	LELCV-[TW-DC-1-Pk-16]	MN692224	
Pumpkin yellow mosaic Malaysia virus MP1 isolate of pumpkin from Malaysia	PuYMMYV-[MY-MP1-Pk-01]	EF197941	
Loofa yellow mosaic virus luffa isolate from Vietnam	LYMV-[VN-Luf]	AF509739	AF509740
Squash leaf curl virus	SLCV-[USA]	M38183	M38182

Table S3. Sequence characteristics of cucurbit-infecting begomovirus DNAs identified in this study.

Island	Province	Virus isolates	DNA-A ORF regions, nt									DNA-B ORF regions, nt				
			Length, nt	IR	AV2	AV1	AC1	AC2	AC3	AC4	Accessions	Length, nt	IR	BV1	BC1	Accessions
	Quezon	SLCCNV-A[PH-Pk38-18]	2752	2601-135	136-474	296-1066	2600-1515	1612-1208	1473-1063	2443-2267	OP771610					
		SLCuPV-A[PH-Pk38-18]										2719	2182-475	476-1282	2181-1336	OP771615
	Cavite	SLCuPV-A[PH-Pk62-18]	2765	2601-135	136-474	296-1066	2600-1515	1612-1208	1473-1063	2443-2186	OP771541	2720	2182-475	476-1282	2181-1336	OP771576
		SLCuPV-A[PH-BoG67-18]	2748	2584-118	119-457	279-1049	2583-1519	1595-1191	1456-1046	2426-2169	OP771542	2719	2182-475	476-1282	2181-1336	OP771577
		SLCuPV-B[PH-Pk76-18]	2746	2584-118	119-457	279-1049	2583-1498	1595-1191	1456-1046	2426-2169	OP771543	2717	2195-474	475-1281	2196-1336	OP771578
	Bulacan	SLCuPV-B[PH-BoG87-18]	2763	2584-118	119-457	279-1049	2583-1498	1456-1046	1595-1191	2426-2169	OP771544	2732	2195-473	474-1280	2194-1334	OP771579
	Nueva Ecija	SLCuPV-B[PH-MM100-18]	2747	2585-119	120-458	280-1050	2584-1514	1457-1047	1596-1192	2427-2170	OP771545	2714	2180-473	474-1280	2179-1334	OP771580
		SLCuPV-B[PH-MM102-18]	2745	2583-117	118-456	278-1048	2582-1497	1594-1190	1455-1045	2425-2168	OP771546	2732	2195-473	474-1280	2194-1334	OP771581
		SLCuPV-B[PH-WM114-18]	2746	2584-118	119-457	279-1049	2583-1498	1595-1191	1456-1046	2426-2169	OP771547	2732	2195-473	474-1280	2194-1334	OP771582
	Nueva Vizcaya	SLCuPV-A[PH-BoG137-18]	2748	2584-118	119-457	279-1049	2583-1498	1595-1191	1456-1046	2426-2169	OP771548	2717	2197-475	476-1282	2196-1336	OP771583
Luzon		SLCCNV-A[PH-Pk140-18]	2752	2601-135	136-474	296-1066	2600-1515	1612-1208	1473-1063	2443-2267	OP771611					
		SLCuPV-A[PH-Pk140-18]										2721	2197-475	476-1282	2196-1336	OP771616
		SLCuPV-A[PH-Pk151-18]	2734	2584-118	119-457	279-1049	2583-1498	1595-1191	1456-1046	2426-2169	OP771549	2719	2198-475	476-1282	2197-1337	OP771584
	Ilocos Sur	SLCuPV-A[PH-BoG183-18]	2736	2585-118	120-458	280-1050	2584-1499	1596-1192	1457-1047	2427-2170	OP771550	2693	2153-446	447-1253	2152-1307	OP771585
		SLCCNV-A[PH-Pk195-18]	2752	2601-135	136-474	296-1066	2600-1515	1612-1208	1473-1063	2443-2267	OP771612					
		SLCuPV-A[PH-Pk195-18]										2640	2114-392	393-1199	2113-1253	OP771617
	La Unión	SLCuPV-B[PH-BoG216-18]	2743	2584-118	119-457	279-1049	2583-1498	1595-1191	1456-1046	2426-2169	OP771551	2703	2181-475	476-1282	2180-1335	OP771586
	Pangasina	SLCuPV-A[PH-BoG227-18]	2747	2584-118	119-457	279-1049	2583-1498	1595-1191	1456-1046	2426-2169	OP771552	2712	2180-474	475-1281	2179-1334	OP771587
		SLCuPV-A[PH-BoG237-18]	2747	2584-118	119-457	279-1049	2583-1498	1595-1191	1456-1046	2426-2169	OP771553	2705	2181-475	476-1282	2180-1335	OP771588
	Tarlac	SLCuPV-A[PH-Pk247-18]	2747	2584-118	119-457	279-1049	2583-1498	1595-1191	1456-1046	2426-2169	OP771554	2713	2180-474	475-1281	2179-1334	OP771589
		SLCuPV-A[PH-Pk248-18]	2749	2584-118	119-457	279-1049	2583-1498	1595-1191	1456-1046	2426-2169	OP771555	2713	2181-475	476-1282	2180-1335	OP771590
	Negros Occidental	SLCCNV-A[PH-Pk11-19]	2752	2601-135	136-474	296-1066	2600-1515	1612-1208	1473-1063	2443-2267	OP771613					
		SLCuPV-A[PH-Pk11-19]										2722	2179-472	473-1279	2178-1333	OP771618
		SLCuPV-A[PH-Pk12-19]	2750	2592-120	121-459	281-1051	2591-1500	1597-1193	1458-1048	2428-2171	OP771556	2724	2181-474	475-1281	2180-1335	OP771591
Visayas		SLCuPV-A[PH-BoG19-19]	2764	2601-135	136-474	296-1066	2600-1515	1612-1208	1473-1063	2443-2186	OP771557	2717	2176-469	470-1276	2175-1330	OP771592
	Iloilo	SLCuPV-A[PH-BoG54-19]	2748	2584-118	119-457	279-1049	2583-1498	1595-1191	1456-1046	2426-2169	OP771558	2723	2182-474	475-1281	2181-1336	OP771593
		SLCuPV-A[PH-BoG56-19]	2748	2584-118	119-457	279-1049	2583-1498	1595-1191	1456-1046	2426-2169	OP771559	2720	2180-473	474-1280	2179-1334	OP771594
		SLCuPV-A[PH-Pk71-19]	2749	2585-119	120-458	280-1050	2584-1499	1596-1192	1457-1047	2427-2170	OP771560	2723	2181-474	475-1281	2180-1335	OP771595
	Davao del Sur	SLCuPV-A[PH-Pk98-19]	2748	2584-118	119-457	279-1049	2583-1498	1595-1191	1456-1046	2426-2169	OP771561	2721	2182-476	477-1283	2181-1336	OP771596
		SLCuPV-A[PH-Pk117-19]	2747	2583-117	118-456	278-1048	2582-1497	1594-1190	1455-1045	2425-2168	OP771562	2721	2181-475	476-1282	2180-1335	OP771597
		SLCuPV-A[PH-Cyt123-19]	2722	2584-118	119-457	279-1049	2583-1498	1595-1191	1456-1046	2426-2169	OP771563	2722	2181-475	476-1285	2180-1335	OP771598
		SLCuPV-A[PH-Pk125-19]	2748	2584-118	119-457	279-1049	2583-1498	1595-1191	1456-1046	2426-2169	OP771564	2714	2175-469	470-1276	2174-1329	OP771599
		SLCuPV-A[PH-Cyt131-19]	2748	2584-118	119-457	279-1049	2583-1498	1595-1191	1456-1046	2426-2169	OP771565	2721	2181-475	476-1282	2180-1335	OP771600
	Bukidnon	SLCuPV-A[PH-Pk166-19]	2748	2584-118	119-457	279-1049	2583-1498	1595-1191	1456-1046	2426-2169	OP771566	2723	2182-476	477-1283	2181-1336	OP771601
		SLCuPV-A[PH-Pk169-19]	2747	2584-118	119-457	279-1049	2583-1498	1595-1191	1456-1046	2426-2169	OP771567	2724	2183-477	478-1284	2182-1337	OP771602
Mindanao		SLCuPV-A[PH-Pk186-19]	2748	2584-118	119-457	279-1049	2583-1498	1595-1191	1456-1046	2426-2169	OP771568	2723	2182-476	477-1283	2181-1336	OP771603
		SLCuPV-A[PH-Pk187-19]	2748	2584-118	119-457	279-1049	2583-1498	1595-1191	1456-1046	2426-2169	OP771569	2722	2181-475	476-1282	2180-1335	OP771604
		SLCuPV-A[PH-Pk205-19]	2748	2584-118	119-457	279-1049	2583-1498	1595-1191	1456-1046	2426-2169	OP771571	2724	2183-477	478-1284	2182-1337	OP771605
		SLCuPV-A[PH-Pk208-19]	2748	2584-118	119-457	279-1049	2583-1498	1595-1191	1456-1046	2426-2169	OP771572	2721	2181-475	476-1282	2180-1335	OP771606
		SLCuPV-A[PH-Pk211-19]	2750	2586-120	121-459	281-1051	2585-1500	1597-1193	1458-1048	2428-2171	OP771573	2722	2181-475	476-1282	2180-1335	OP771607
		SLCuPV-A[PH-Pk212-41-19][a]	2747	2583-117	118-456	278-1048	2582-1497	1594-1190	1455-1045	2425-2168	OP771574	2724	2183-477	478-1284	2182-1337	OP771608
		SLCCNV-A[PH-Pk212-42-19][a]	2741	2586-120	121-459	281-1051	2585-1500	1597-1193	1458-1048	2428-2252	OP771614					
		SLCuPV-A[PH-Pk212-42-19]										2724	2183-477	478-1284	2182-1337	OP771619
		SLCuPV-A[PH-Cyt231-19]	2748	2584-118	119-457	279-1049	2583-1498	1595-1191	1456-1046	2426-2169	OP771575	2723	2182-476	477-1283	2181-1336	OP771609

[1] The begomovirus DNA-B was identified in same sample. SLCCNV: *Squash leaf curl China virus*; SLCuPV: *Squash leaf curl Philippines virus*.
